# The association between childhood motor performance and developmental trajectories of sport participation over 5 years in Danish students aged 6–16-year-old

**DOI:** 10.1038/s41598-023-31344-x

**Published:** 2023-03-13

**Authors:** Charlotte Raadkjær Lykkegaard, Helene Støttrup Andersen, Sonja Wehberg, Sinead Holden, Frans Boch Waldorff, Jens Søndergaard, Lisbeth Runge Larsen, Heidi Klakk, Niels Wedderkopp

**Affiliations:** 1 The Research Unit of General Practice, Department of Public Health, University of Southern Denmark, J.B Winsloews Vej 9, 5230 Odense M, Denmark; 2 Centre for General Practice at Aalborg University, Aalborg University, 9220 Åalborg East, Denmark; 3 Section of General Practice and The Research Unit for General Practice, Department of Public Health, University of Copenhagen, 1353 Copenhagen, Denmark; 4 Department of Health Science and Technology, Aalborg University, 9220 Åalborg East, Denmark; 5 UCD Clinical Research Centre, School of Medicine, University College Dublin, Dublin 4, Ireland; 6 Center for Research in Childhood Health, Department of Regional Health Research, University of Southern Denmark, 5230 Odense, Denmark; 7 Head of Studies, Education and Social Education Svendborg, UCL University College, 5700 Svendborg, Denmark; 8 Centre for Clinical Research and Prevention, Section for Health Promotion and Prevention, Bispebjerg- and Frederiksberg Hospital, 2000 Frederiksberg, Denmark; 9 Exercise Epidemiology, Institute of Sports science and Biomechanics, University of Southern Denmark, Odense M, Denmark

**Keywords:** Epidemiology, Paediatric research

## Abstract

Sports participation has potential to promote physical activity in youth. Unfortunately, sports participation and physical activity may decline from childhood to adolescence and into adulthood. Globally, only 20% of 13–15-year-olds meet the World Health Organisation recommendations for physical activity. This study aimed to investigate the 5-year trajectories of sports participation and their association with baseline motor performance in Danish school children as part of the Childhood Health Activity and Motor Performance School Study-Denmark (CHAMPS-DK), a school-based physical activity intervention study which investigated the health benefits of increased physical education lessons. Five distinct trajectories were identified, with group 1 maintained a stable trajectory of little to no sports participation, and group 2 showing a low decreasing trend. Group 3–5, the most sports active, demonstrated increasing sport participation at different rates. Baseline motor performance score was associated with the two most active sports participation groups. Students who were more physically active during school hours participated less in organised leisure time sports. This suggest focusing on improving motor performance in youth may support future sports participation and thus health-related physical activity. But also, that it might be necessary to engage and maintain children and adolescents in leisure time sports while implementing physical activity promotion interventions.

## Introduction

Sports participation is considered an important motivator for promoting physical activity in youth^3^. Several studies have consistently shown that children and adolescents who participate in organized sports clubs during the week are more likely to meet the physical activity recommendation^1–7^ and have a higher social competence, well-being, physical fitness, and health profile compared to those who do not participate in organized sports^8–11^. Unfortunately, sports participation and daily physical activity decreases from childhood to adolescence, girls at a younger age than boys^1,2^. The World Health Organisation (WHO) recommends at least 60 min of moderate- to vigorous-intensity physical activity (MVPA) daily for 10–17-year-olds^12^ to achieve numerous health benefits^13^. Globally, only 20% of 13–15-year-olds meet this recommendation^3,4,14^ and in Danish school students aged 6–11-years-old, 11–68% depending upon age fail to reach the recommended levels^1^, representing a significant public health challenges.

Previous cross-sectional studies have linked good motor performance to being more physical- and sports active^15^, and vice versa^16–18^. However, details on the effect of childhood motor performance on the development of sports participation during adolescence remains unclear.

The Childhood Health Activity and Motor Performance School Study-Denmark (The CHAMPS-study DK)^19^ provides a unique opportunity to generate detailed knowledge of children`s sports participation development during a 5-year period and link it to baseline motor performance.

Group-based trajectory modelling uses distinct developmental trajectories to identify subgroups of individuals whit similar development patterns.

This study aims to describe the individual developmental trajectories of sports participation over five years in Danish children aged 6–15 years-old, identifying distinctive subgroups of individual trajectories within the population, and the distribution of sex, school type, and sports type within the subgroups. Furthermore, this study aims to assess the association between fundamental baseline motor performance and future leisure time sport participation, using distinctive trajectories of sport participation as an outcome.

## Methods

### Study design

The present study is a secondary analysis that is nested within the CHAMPS-study DK^19,20^, which was designed as a longitudinal quasi-experimental trial to investigate the health benefits of increased physical education lessons. The CHAMPS-study DK was initiated as a part of a community project, “The Svendborg project”, which started in 2008 and included school students aged 6–15 years from the municipality of Svendborg, Denmark. All participants were monitored weekly over a 5-year period, from October 2008 to June 2014. It should be noted that this secondary analysis was not a part of the original aim of the CHAMPS-study DK, and the original experimental design was not included in this analysis.

### Setting

All 19 primary schools in the municipality of Svendborg, which has a population of 58.600, were invited to participate in the CHAMPS-study Dk. Six schools agreed to participate as sports schools, while four schools were included as control schools. The control schools were matched to the sports schools based on the size and socio-economic groups within their uptake areas. The six intervention schools provided an additional four physical education lessons, totalling 270 min per week, for all students from pre-school (age 5) to the sixth grade (age 12), while the four control schools continued to provide the mandatory two physical education programme in Denmark, which lasted 90 min per week^21^. Further information on the study sample and procedures have been previously reported^19–21^.

### Participants

All children from grades one to five (age 5–10) and their parents from the participating primary schools were invited to participate in the study. Children who provided consent were included consecutively from November 2008 to June 2009. As the study followed a natural experimental design, new children were allowed to enter the study and all children were allowed to leave at any time. Children with chronic diseases were not included in the study.

### Data

We analysed data from baseline (Nov 2008–June 2009) through a 5-year follow-up period from August 2009 to June 2014, combining data from the sports schools and the control schools into a common cohort. At baseline, all children underwent physical testing and completed questionnaires with their parents. Over the 5-years period, the children were monitored through weekly questionnaire, with the exception of the Christmas holidays and the six-weeks summer holidays. Due to the natural experimental design of the CHAMPS-study DK, children who moved away from the area left the study, while new children who moved into the area were added to the study.

#### Sports participation

Parents reported their child´s participation in organized leisure-time sports using a mobile phone application called SMS-track (https://sms-track.com/). Each week, parents received an automated text message asking how many times their child had participated in organized leisure-time sports during the preceding week (0–7 or 8). The response options ranged from 0 to 7, with the option of selection 8 indicating that the child had participated more than seven times in any organized leisure-time sport.

If a number between 1 and 8 was reported, an additional question was sent asking the specific type of sports the child participated in. Ten options were suggested based on the most popular sport types in the local community: 1—soccer; 2—handball; 3—basketball; 4—volleyball; 5—rhythmic gymnastics; 6—tumbling gymnastics; 7—swimming; 8—horse-riding; 9—dancing; and 10—other sports. Parents were able to report more than one sports type each week. All responses were automatically recorded into a secure database.

If more than one sports type was reported and the reported number of sessions exceeded the number of reported sport types, we allocated the reported number of sport sessions equally between the reported sport types.

#### Motor performance

Motor performance measures were collected by trained research staff at baseline in school gyms or sports halls. The research staff underwent two full days of practice, including standardised calibration of the equipment, measurement, and instruction procedures. They practiced on each other tested the procedures on children of the same age as the CHAMPS-study DK participants^19,22^. Six validated tests were used to assess motor performance, including:

1. Backward balance from the Körperkoordinations Test für Kinder^23,24^, a valid and reliable test battery^25^. Participants walked backward on three different balance beams (6, 4.5, and 3 cm wide) with three trials on each beam, and the number of successful footsteps was recorded, with a maximum of eight points per trial. Possible scores ranged from 0 to 72 points.
2. Precision throw from Der Allgemeiner Sportmotorischer Test für Kinder von 6–11 Jahren^26^. Participants stood 3 m from a target plate and had two sets of five throws with a tennis ball. Each throw was scored 0–3 point, with a maximum of 30 points.
3. Hand grip strength from the Eurofit test battery^27^, measured in kilograms using the JAMAR dynamometer (Scandidact, Cat. No. 281128). The best of two trials was recorded. This test is found valid for assessing upper body maximal strength^28^.
4. Vertical jump test, corresponding to the Abalakow vertical jump test (belt test)^29^. This test is a valid proxy for strength in the lower extremity and is measured as jumping height in centimetres. The belt tests provide a more objective measure of vertical-jump performance compared with the traditional jump and reach tests^29^.
5. Shuttle run from the Eurofit test battery^27^, is a agility test and is valid to estimate cardiorespiratory fitness^28^. Participants completed five laps on a 5-m lane, and the time was measured in seconds.
6. The Andersen test is a test of cardiorespiratory fitness, measured as the number of meters run in an intermittent shuttle run test^30^. This test is found reproducible and can be used as an indicator of aerobic fitness/performance for children and adolescents^30,31^.

### Statistical analyses

Group-based trajectory modelling^32^ was used to identify distinct developmental trajectories of sports participation from age 6 to 16, measured as average weekly sports participation.

To run our analyses, we calculated average weekly sports participation per month referred to as average weekly sports participation. For each child, the sum of weekly sports sessions in a month was divided by the number of corresponding weeks with available observations. In total 129 time points (months) were included in the analyses. The weekly sports participation was considered missing when there were two weekly measurements or less per a given month. If participants had only one calculated monthly measurement, they were excluded. The corresponding monthly age of the children was calculated.

To estimate the group-based trajectory models, we used the Stata Plugin traj^33^ for a censored normal outcome (option cnorm) with the minimum and maximum (censoring) set to respectively 0 and 8. We fitted the fullest polynomial model possible in the plugin, allowing for linear, quadratic and cubic effects. Missing data was handled by Traj using the assumption that the data is missing completely at random(MCAR)^34^.

To identify a suitable number of groups, we looked at Akaike Information Criterion (AIC), Bayesian Information Criterion (BIC), the Average Posterior Probability of Assignment (APPA) and the related Odds of Correct Classification (OCC)^35^ as well as the minimum number of children assigned to a group. Both APPA and OCC are defined by group. We aimed for an APPA above 0.7 and an OCC above 5 for all groups. In addition, we employed *k*-fold cross-validation where we randomly split the dataset (on the child level) into *k* parts, fitted the model on *k*-1 parts and evaluated the fit (prediction) on the remaining *k*th part of the data^36^. We chose *k* as 2, 10 and 50 as well as leave-one-out (LOO) cross-validation where each observation (child) was left out in turn. We used one random split of the data for *k* = 2, 10 and 50.

To estimate the effect of baseline motor performance on future sport participation, we used a multinomial regression model with groups of trajectories as outcome and motor performance, sex, and school type as explanatory factors. Possible effects of clustering on schools, and classes were considered by using the robust variance estimator^37,38^, and not by modelling a specific random effect. Our approach acknowledges the data as clustered while keeping the property that univariable regression estimates coincide with the directly calculable relative risk ratios.

We divided the baseline motor performance scores into a health-related fitness score (including the hand grip test and the Andersen test) and a coordination-related fitness score (including vertical jump, shuttle run, backward balance, and precision throw) and a total score according to previous studies^18,39^.

To calculate the total baseline motor performance scores we used a sex- and age stratified z-score taking into account that the students were included in the study at different ages and that ability of motor performance biologically changes with sex and age^40^. Furthermore, we divided the motor performance scores into tertiles low, middle, high.

All analyses were performed using the software StataCorp. 2021. (*Stata Statistical Software: Release 17*. *College Station, TX: StataCorp LLC*). The significance level was 5%.

### Ethics

The study was performed in accordance with the Declaration of Helsinki and reported to SDU Research & Innovation Organisation (Notification number 10.880). The CHAMPS-study DK was approved by the Regional Scientific Ethical Committee of Southern Denmark (ID S-20080047) and registered with the Danish Data Protection Agency (J.nr. Physical Activity Responses in Children 2255 2008-41-2240).

Prior to the enrolment in the study parents provided written informed consent and all children gave verbal consent. All participation was voluntary with the option to withdraw at any time.

## Results

This study included 1553 school students, consisting of 801 girls and 752 boys. Six students were excluded from this study due to their limited participating (less than two months), leaving 1547 students for the trajectory analysis (Table 1). As data collection was suspended during holidays and due to the open study design, the participants´ total participation varied over time, and in total they provided data for up to 4 years and 3 months during the 5-year follow up period.

**Table 1:** Descriptive characteristics of this study’s participants by age (years) at start of study participation.

Age (years) at start of study participation	6–7	8–9	10–11	12-13^a^	All
All n (%)	256 (16.5)	549 (35.4)	595 (38.3)	152 (9.8)	1547 (100)
Sex					
Girls	144 (56.7)	277 (50.5)	300 (50.6)	77 (51.0)	798 (51.6)
Boys	110 (43.3)	272 (49.5)	293 (49.4)	74 (49.0)	749 (48.4)
School type					
Control	102 (40.2)	235 (42.8)	257 (43.3)	71 (47.0)	665 (43.0)
Sports schools	152 (59.8)	314 (57.2)	336 (56.7)	80 (53.0)	882 (57.0)
Motor performance population					
All n (%)	246 (22.2)	449 (40.5)	391 (35.3)	23 (2.1)	1109 (100)
Sex					
Girls	141 (57.3)	223 (49.7)	202 (51.7)	12 (52.2)	578 (52.1)
Boys	105 (42.7)	226 (50.3)	189 (48.3)	11 (47.8)	531 (47.9)
School type					
Control	100 (40.7)	191 (42.5)	172 (44.0)	12 (52.2)	475 (42.8)
Sports schools	146 (59.3)	258 (57.5)	219 (56.0)	11 (47.8)	634 (57.2)

^a^Including one child aged 14.

We had a total of 245,703 observations and 401,955 numbers of sport sessions. For further descriptive details on sports participation, please refer to Table S1 and S2.

**Figure 1:**
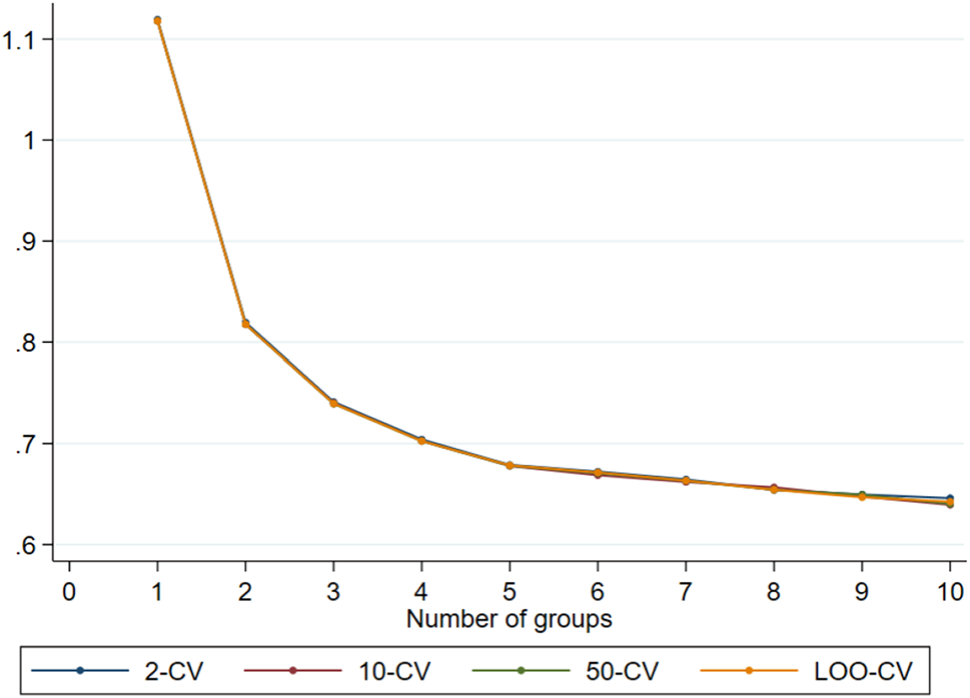
Estimated mean distance for 2-CV, 10-CV, and 50-CV over number of groups.

To identify the suitable number of groups for the analysis of group-based trajectories, we examined various models using different criteria. As expected, the AIC and BIC tended to favour larger models, as shown in Table 2: However, based on the cross-validation, models 5 and 6 were identified as appropriate models, as shown in Fig. 1. The decrease in mean stabilized at five groups, indicating that using more than five groups does not improve the result. Therefore, we selected the model with five groups as the best model, as it ensures a sufficient number of students in all groups (Table 2 and Fig. 1). For more information on models including four, five and six groups, please refer to supplementary table S4.

**Table 2:** Indices used for determining a suitable number of groups.

N of groups	N of variables	AIC	BIC (obs)	BIC (children)	APPA (min)	OCC (min)	2-CV	10-CV	50-CV	LOO-CV	N of children (min)
1	5	− 107,128	− 107,151	− 107,142	1.00	−	1.12	1.12	1.12	1.12	1547
2	10	− 93,611	− 93,656	− 93,638	0.99	62.26	0.82	0.82	0.82	0.82	668
3	15	− 88,648	− 88,716	− 88,688	0.97	39.61	0.74	0.74	0.74	0.74	332
4	20	− 86,234	− 86,324	− 86,287	0.96	40.34	0.70	0.70	0.70	0.70	152
5	25	− 85,027	− 85,140	− 85,094	0.94	43.89	0.68	0.68	0.68	0.68	133
6	30	− 84,284	− 84,420	− 84,365	0.94	42.69	0.67	0.67	0.67	0.67	14
7	35	− 83,747	− 83,905	− 83,841	0.90	31.70	0.66	0.66	0.66	0.66	14
8	40	− 83,282	− 83,462	− 83,389	0.88	37.26	0.65	0.66	0.65	0.65	9
9	45	− 82,859	− 83,062	− 82,979	0.85	40.35	0.65	0.65	0.65	0.65	10
10	50	− 82,469	− 82,695	− 82,603	0.82	34.92	0.65	0.64	0.64	0.64	10

The full models were fitted based on N = 61,398 observations on 1547 children.

AIC = Akaike Information Criterion; APPA = Average Posterior Probability of Assignment; BIC = Bayesian Information Criterion; OCC = Odds of correct classification; K-CV = we employed k-fold cross-validation where we randomly split the dataset (on the child level) into k parts, fitted the model on k − 1 parts and evaluated the fit (prediction) on the remaining kth part of the data(33). We chose k as 2, 10 and 50 as well as leave-one-out (LOO-CV) cross-validation where each observation (child) was left out in turn. We used one random split of the data for k = 2, 10 and 50.

**Figure 2:**
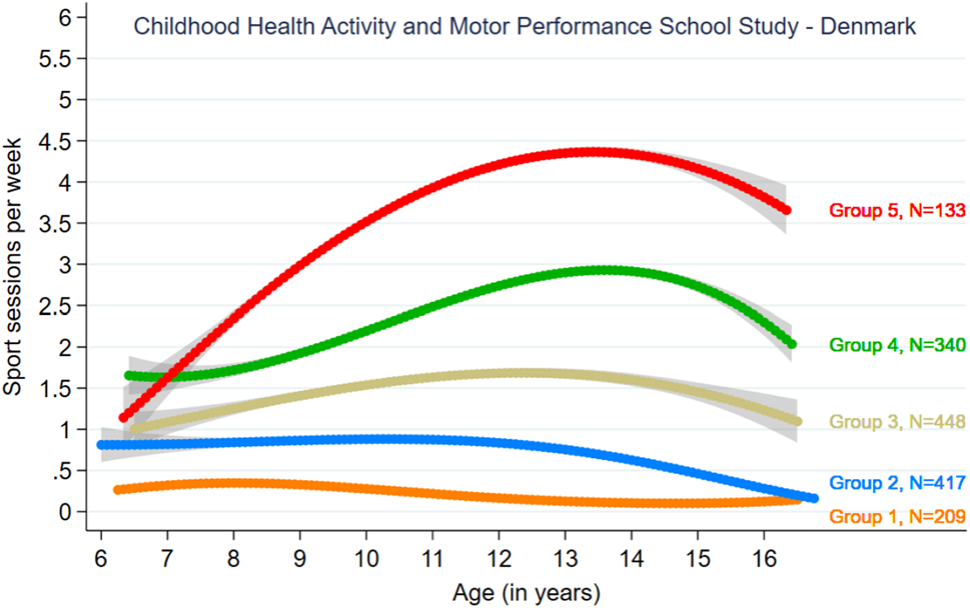
Estimated group-based trajectories with 95% point-wise confidence intervals (shown as bands) for model 6G.

In the model with five groups (Table 3 and Fig. 2), the group size ranged from 133 to 448 students (Fig. 2). The corresponding numbers used to generate Fig. 2 can be found in supplementary table S5. We observed a major difference in the average weekly sports participation between the five groups with distinct trajectories over the 5-year period. Group 1, the “non-sports participating student,” had consistently low or no sports participation over five years, with an average weekly sports participation of less than 0.40 sessions. Group 2 had low sports participation, which decreased to almost no sports participation over time (‘low decreasing sports participation’).

**Table 3:** Group distribution by sex, school type, motor performance and sports type for the trajectory model including 5 groups.

Sports participation trajectory groups	Group 1	Group 2	Group 3	Group 4	Group 5	Overall
Sports participation						
All n	209 (13.5)	417 (27.0)	448 (29.0)	340 (22.0)	133 (8.6)	1547 (100)
Sex n (%)						
Girls	97 (12.2)	259 (32.5)	227 (28.4)	150 (18.8)	65 (8.1)	798 (100)
Boys	112 (15.0)	158 (21.1)	221 (29.5)	190 (25.4)	68 (9.1)	749 (100)
School type n (%)						
Control	70 (10.5)	153 (23.0)	204 (30.7)	174 (26.2)	64 (9.6)	665 (100)
Sport schools	139 (15.8)	264 (29.9)	244 (27.7)	166 (18.8)	69 (7.8)	882 (100)
Total motor performance^b^ at baseline^a^ n (%)						
All n	145	284	314	263	103	1109 (100)
Low	67 (46.2)	108 (38.0)	106 (33.8)	72 (27.4)	18 (17.5)	371 (33.5)
Middle	50 (34.5)	99 (34.9)	103 (32.8)	83 (31.6)	35 (34.0)	370 (33.4)
High	28 (19.3)	77 (27.1)	105 (33.4)	108 (41.1)	50 (48.5)	368 (33.2)
Health-related motor performance^c^ at baseline^a^ n (%)						
Low	63 (43.4)	117 (41.2)	99 (31.5)	68 (25.9)	24 (23.3)	371 (33.5)
Middle	45 (31.0)	96 (33.8)	104 (33.1)	92 (35.0)	33 (32.0)	370 (33.4)
High	37 (25.5)	71 (25.0)	111 (35.4)	103 (39.2)	46 (44.7)	368 (33.2)
Coordination-related motor performance^d^ at baseline^a^ n (%)						
Low	67 (46.2)	104 (36.6)	105 (33.4)	74 (28.1)	21 (20.4)	371 (33.5)
Middle	47 (32.4)	104 (36.6)	103 (32.8)	85 (32.3)	31 (30.1)	370 (33.4)
High	31 (21.4)	76 (26.8)	106 (33.8)	104 (39.5)	51 (49.5)	368 (33.2)
Mean sports sessions per group member, n (%)						
All	33 (100)	122 (100)	254 (100)	420 (100)	623 (100)	257 (100)
Soccer	4.5 (13.6)	20.3 (16.6)	78.1 (30.7)	152.0 (36.2)	181.4 (29.1)	77.7 (30.2)
Handball	3.0 (9.09)	10.8 (8.9)	47.2 (18.6)	112.2 (26.7)	159.1 (25.5)	55.3 (21.5)
Swimming	5.3 (16.06)	17.1 (14.02)	17.4 (6.9)	20.5 (4.9)	51.7 (8.3)	19.3 (7.5)
Horseback riding	1.9 (5.8)	6.5 (5.3)	13.8 (5.4)	30.8 (7.3)	108.7 (17.4)	22.1 (8.6)
Rythm gymnastic	2.4 (7.3)	6.1 (5.00)	8.6 (3.4)	11.3 (2.7)	13.1 (2.1)	8.1 (3.2)
Tumbling gymnastic	0.5 (1.5)	3.8 (3.1)	6.3 (2.5)	8.2 (2.0)	2.3 (0.4)	4.9 (1.9)
Basketball	0.5 (1.5)	2.1 (1.7)	8.6 (3.4)	4.2 (1.00)	7.0 (1.1)	4.7 (1.8)
Volleyball	3.4 (10.3)	10.5 (8.6)	15.0 (5.9)	28.3 (6.7)	35.5 (5.7)	16.9 (6.6)
Dance	1.4 (4.2)	9.2 (7.5)	9.7 (3.8)	6.3 (1.5)	5.7 (0.9)	7.3 (2.8)
Others	10.0 (30.3)	35.8 (29.3)	49.8 (19.6)	46.3 (11.02)	58.6 (9.4)	40.6 (15.8)

Group 1 is the subgroup of individuals with the lowest activity level and group 5 is the subgroup of individuals with the highest activity level.

The z-score was multiplied by -1 if a better performance meant a lower values. The health-, coordination- and total score were then calculated by summing the relevant variables z-scores and then divided by the number of included variables.

^a^Motor performance scores were based on calculated z-scores. Z-score = (variable value – mean of values)/SD.

^b^Including all 6 motor performance tests.

^c^Including the hand grip test and the Andersen test.

^**d**^Including vertical jump, shuttle run, backward balance, and precision throw.

In contrast, groups 3–5 were the most sports active and increased their sport participation at different rates. Group 3 had relatively low sports participation with only a slight increase, whereas group 4 had moderate increasing sports participation. Group 3 experienced a slight decrease in sports participation from age 13 to 16, and group 4 had a moderate decrease from age 14 to 16. Group 5 (“rapidly increasing high sport participation”) showed the most significant increase in sports participation from age 6 to age 13–14, reaching up to 4.4 sessions per week, followed by a moderate decrease, and ending at 3.5 sessions per week (Fig. 2).

The most commonly represented sport type across all groups was handball (8.9–26.7%). Additionally, the category “Others” (a mix of uncategorised sport types) was highly represented in all groups (9.4–30.3). Swimming, volleyball, and other sports had the highest proportion in the less active groups, while handball and soccer had the highest proportion in the most active groups (Table 3 and S2). Furthermore, both genders were rather equally represented across the five trajectory groups (Table 3).

Due to incomplete motor performance data, 68–70% of participants in trajectory group 1 to 3 were included, while 77.4% of participants in trajectory group 4 and 5 were included (Table 4).

**Table 4:** Numbers of participants included in our trajectory analysis and our analysis including motor performance.

	Group 1	Group 2	Group 3	Group 4	Group 5	Overall
Model 5G						
Traj analysis (n)	209	417	448	340	133	1547
Motor performance analysis (n)	145	284	314	263	103	1109
Percent (%)^a^	69.4	68.1	70.1	77.4	77.4	71.7

^a^Indicates how many percent of the participants in each group are included in our regression analysis.

Furthermore, participants with higher baseline motor performance scores were more likely to belong to higher the sports participation groups (Table 3).

The regression analysis, which included motor performance data, was conducted on data from 1109 school students (Table 4). The multinomial regression results indicated a positive association between a high total motor performance z-score at baseline and future sports participation for the two most active trajectory sports groups (group 4–5) (RRR 3.40, CI 2.04–5.68; RRR 6.83, CI 3.37–13.83) compared to a low total motor performance at baseline (Table 5).

**Table 5:** Results from multinomial logistic regression of the sports participation trajectory groups and gender, school type, and motor performance.

	Group 1	Group 2		Group 3		Group 4		Group 5		Overall *p* value
		RRR	CI (95%)	RRR	CI (95%)	RRR	CI (95%)	RRR	CI (95%)	
Participants n = 1547										< 0.001**
*Gender* (ref girls)	Ref	0.53	(0.35–0.80)**	0.76	(0.51–1.13)	0.95	(0.63–1.44)	0.71	(0.42–1.18)	
*School type* (ref control)	Ref	0.83	(0.55–1.27)	0.66	(0.44–1.00)*	0.55	(0.36–0.84)**	0.53	(0.31–0.89)*	
Total motor performance z-score at baseline n = 1109										0.000**
*School type* (ref control)	Ref	0.83	(0.55–1.27)	0.65	(0.43–0.98)*	0.52	(0.34–0.79)**	0.48	(0.29–0.82)**	
Motor performance^b^ z-score^a^										
Low	Ref	1.00		1.00		1.00		1.00		
Middle	Ref	1.79	(1.11–2.87)*	1.52	(0.95–2.44)	2.18	(1.32–3.61)**	4.06	(2.00–8.25)**	
High	Ref	1.56	(0.94–2.58)	1.87	(1.14–3.06)	3.40	(2.04–5.68)**	6.83	(3.37,13.83)**	
Motor performance score divided in health- and coordinated related z-score at baseline										0.000**
*School type* (ref control)	Ref	0.79	(0.51–1.21)	0.64	(0.42–0.97)*	0.50	(0.32–0.77)**	0.45	(0.26–0.79)**	
Health-related motor performance^c^ z-score^a^										
Low	Ref	1.00		1.00		1.00		1.00		
Middle	Ref	1.10	(0.67–1.82)	1.52	(0.92–2.50)	1.85	(1.10–3.11)*	1.28	(0.65–2.51)	
High	Ref	0.75	(0.43–1.31)	1.46	(0.85–2.52)	1.59	(0.90–2.81)	1.41	(0.69–2.87)	
Coordination-related motor performance^d^ z-score^a^										
Low	Ref	1.00		1.00		1.00		1.00		
Middle	Ref	1.40	(0.85–2.31)	1.22	(0.74–2.00)	1.44	(0.85–2.42)	2.05	(1.02–4.12)**	
High	Ref	1.98	(1.11–3.53)*	1.63	(0.92–2.89)	2.52	(1.40–4.56)**	5.09	(2.36–10.97)**	

Group 1 is the subgroup of individuals with the lowest activity level and group 5 is the subgroup of individuals with the highest activity level.

*p > 0.05, **p > 0.01 compared the sports participation trajectory groups with gender, school type, and motor performance.

^a^Total motor performance z-score is stratified for age in categories at the time of motor performance baseline test. Z-score = (variable value − mean of values)/SD.

^b^Including all 6 motor performance tests.

^c^Including the hand grip test and the Andersen test.

^d^Including vertical jump, shuttle run, backward balance, and precision throw.

The representation of students who attended a sports school was significantly lower in trajectory groups 3 -5 (RRR 0.66, CI 0.44–1.00; RRR 0.55, CI 0.36–0.84; RRR 0.53, CI 0.31–0.89) compared to the reference group, as shown in Table 5.

## Discussion

The study identified five distinct sports participation trajectory groups, with varying rates of increase and decrease in sports participation over the 5-year period. Group 1 showed very little sports activity, while group 2 had low and decreasing sports participation. Group 3–5 showed an increasing trend in sports participation, with group 5 having the highest level of sports activity.

Interestingly, students who had extra physical education (PE) in school participated less in organised sport in their leisure time, which is concerning considering the drop in sports participation after age 14 and the fact that many stop doing sports during the transition to young adulthood^41^. While extra PE has several health benefits especially for the nonhealthy students^13,42–45^, it may be warranted that physical activity interventions in school are followed by an efforts to encourage children and adolescents to engage in leisure time sports that they can continue after leaving school.

We found a significant positive association between baseline motor performance and the two trajectory groups including the most sports active students (group 4 and 5), after controlling for age, gender, and school type. This supports previous cross-sectional studies that have investigating the relationship between current fundamental motor performance and sports participation^46–48^. These findings suggest that focusing on improving motor performance from a young age may increase the likelihood of future participation in leisure time sports, and thereby promote health-related physical activity in primary school students. While genetic and epigenetic factors may play a role in predisposition to physical performance and fitness^49^, participation in activities that enhance motor performance, such as leisure time sports, may also contribute to increased sports participation in the future^18^. However, in the youngest years we saw the lowest weekly sports participation and it might be so that sports participation and motor performance are interrelated. So, both the inborn and the participation in activities that enhances their motor performance (e.g., leisure time sport) and the combination will probably increase the likelihood of future leisure time sport.

### Methodological considerations

One of the strengths of this study is its unique data material. To our knowledge, no previous studies have had such a truly longitudinal data covering an extended period, as this study does, allowing for the study of individual and group-based trajectories.

Another strength is the use of group-based trajectory analysis, which is a person-centred approach that identifies groups of individuals who share attributes. Compared to variable-centred analyses, group-based trajectory models are well-suited to identify meaningful but unknown homogeneous subgroups that follows distinct trajectories, without the need for additional covariates. This approach allowed us to explore patterns in data and key characteristics of individuals following the distinctive developmental pathways^50^. In this case, we were able to identify meaningful subgroups of school students based on their weekly sports participation over five years and describe characteristics of each group. Additionally, this model also allows for several trajectory shapes, which is useful if one trajectory shape is not assumed to fit all.

There exist several statistical approaches to uncover distinct trajectories in longitudinal repeated measures data^35^. From this pool of methods, we chose to analyse our data by GBTM which is a special case of finite mixture models where development curves are modelled by polynomial functions up to cubic terms. While this approach naturally incorporates participant-wise clustering, it does not, however, offer consider clustering between participants, for example due to schools or classes. In addition, previous analysis on CHAMPS^1,45^ and related studies such as The European Youth Heart Study, Denmark^51^ point to rather negligible effects of clustering in classes on the outcomes such as sports participation or body composition.

The methods used to collect data on sports participation only recorded the number of times students participated, without considering the duration or intensity of each session. While this could introduce bias, we believe that asking parents to estimate the time spent on each practice and match would be equally imprecise. Furthermore, it was easier for participants to remember the number of sessions rather than the time spent as the time spent could vary from day to day.

Another limitation was that, when multiple sport types were reported, we had to divide the reported numbers of sports sessions among the reported sports, which could lead to over- or underestimation of the number of sessions for each sport. However, the exact number of sessions per sports was not critical for our main analysis and did not affect the identification of trajectory groups or the number of participants in each group. Although imputed data were available^52^, we decided to use the original data as the grouped based trajectory model does not require fully observed data.

Previous research has suggested that family cultures may play a role in influencing especially boys´ inclination participate in sports, particularly those who are predisposed to sport^53,54^. However, we were unable to investigate the influence of parents and family culture on sports participation in our study, as we did not collect data on these factors. Nevertheless, our findings indicate that individuals who had more physical activity during school were less likely to participate in leisure time sports. This could suggest that their parents were content with the amount of PA their children were already engaged in during the week, and thus did not encourage further participation in sports during leisure time.

To cover a wide spectre of motor skills we combined motor performance test from different validated tests batteries which could be a limitation in our study. But as the tests cover different skills, we found it acceptable to combine these tests.

One limitation of our study is that our data collection period is not contemporary. However, recent studies on physical activity in children and adolescents support our findings that children and adolescents worldwide do not reach the recommended daily levels of PA and that the level decreases with age.

It remains inconclusive whether there is a declining trend in children´s PA level or not, as estimates vary at global, regional, and national levels^55^. A Norwegian study conducted before the COVID-19 pandemic found that the PA level in Norwegian youth have been fairly stable between 2005, 2011 and 2018^56^. A systematic review concludes that during the pandemic, children and adolescents experienced measurable reductions in physical activity, but this reduction varied greatly globally and depended on family, social, and community support and mechanisms, as well as the corresponding season where restriction coincided^57^. Only time will tell whether this decline is temporary or persistent.

## Conclusion

In conclusion, our study identified five distinct sports participation trajectory groups with varying patterns over time. We found that higher baseline motor performance scores were positively associated with future sports participation, indicating the importance of focusing on motor skill development from early age. However, we also observed a trend that participants who were more physically active at school were less likely to participated in organised sports in their leisure time, which raises concerns for long-term physical activity levels.

Our findings suggest that focusing on improving motor performance in young childhood might be important to support and increase level of sports participation and thereby future health-related physical activity. Additionally, school-based physical activity promotion interventions should aim not only to improve physical activity in school but also engage and maintain children and adolescents participating in leisure time sports.

Implementing motor performance programs in the youngest school students could positively influence their ability, enjoyment, and motivation to participate in leisure time sport. Given that, future studies would do well if able to investigate the effect of motor performance interventions programmes on future sports participation in young school students.

## Supplementary Information

Supplementary Tables.

## Data Availability

Data are available on request from the CHAMPS Study Steering Committee due to legal and ethical restrictions. Interested parties may contact Professor Niels Wedderkopp (nwedderkopp@health.sdu.dk), and the following information will be required at the time of application: a description of how the data will be used, securely managed, and permanently deleted.
