# Author Correction: The association between childhood motor performance and developmental trajectories of sport participation over 5 years in Danish students aged 6–16-year-old

**DOI:** 10.1038/s41598-023-34564-3

**Published:** 2023-05-09

**Authors:** Charlotte Raadkjær Lykkegaard, Helene Støttrup Andersen, Sonja Wehberg, Sinead Holden, Frans Boch Waldorff, Jens Søndergaard, Lisbeth Runge Larsen, Heidi Klakk, Niels Wedderkopp

**Affiliations:** 1 The Research Unit of General Practice, Department of Public Health, University of Southern Denmark, J.B Winsloews Vej 9, 5230 Odense M, Denmark; 2 Centre for General Practice at Aalborg University, Aalborg University, 9220 Åalborg East, Denmark; 3 Section of General Practice and The Research Unit for General Practice, Department of Public Health, University of Copenhagen, 1353 Copenhagen, Denmark; 4 Department of Health Science and Technology, Aalborg University, 9220 Åalborg East, Denmark; 5 UCD Clinical Research Centre, School of Medicine, University College Dublin, Dublin 4, Ireland; 6 Center for Research in Childhood Health, Department of Regional Health Research, University of Southern Denmark, 5230 Odense, Denmark; 7 Head of Studies, Education and Social Education Svendborg, UCL University College, 5700 Svendborg, Denmark; 8 Centre for Clinical Research and Prevention, Section for Health Promotion and Prevention, Bispebjerg- and Frederiksberg Hospital, 2000 Frederiksberg, Denmark; 9 Exercise Epidemiology, Institute of Sports science and Biomechanics, University of Southern Denmark, Odense M, Denmark

Correction to: *Scientific Reports* https://doi.org/10.1038/s41598-023-31344-x, published online 13 March 2023

The original version of this Article contained errors in the spelling of the authors Jens Søndergaard, Lisbeth Runge Larsen and Niels Wedderkopp, which were incorrectly given as Jens Søndergaarda, Lisbeth Runge Larseng and Niels Wedderkoppf.

The original Article has been corrected.

