# Supplementary material for: The association between childhood motor performance and developmental trajectories of sport participation over 5 years in Danish students aged 6–16-year-old
Source: Sci Rep. 2023 Mar 13;13:4133. doi: 10.1038/s41598-023-31344-x (PMC10009835; doi:10.1038/s41598-023-31344-x)
Supplement: Supplementary file 1 — Supplementary Tables. [file 41598_2023_31344_MOESM1_ESM.pdf]

The association between childhood motor performance and developmental trajectories of sport participation over five years in Danish students aged 6-16-year-old

Charlotte Raadkjær Lykkegaard[a*], Helene Støttrup Andersen[a], Sonja Wehberg[a], Sinead Holden[b,d,e] Frans Boch Waldorff [a,c], Jens Søndergaard[a], Lisbeth Runge Larsen[g], Heidi Klakk[f,h] , Niels Wedderkopp[f]

[a] The Research Unit of General Practice, Department of Public Health, University of Southern Denmark, DK-5230 Odense, Denmark

[b] Centre for General Practice at Aalborg University, Aalborg University, 9220 Aalborg East, Denmark

[c] Section of General Practice and The Research Unit for General Practice, Department of Public Health, University of Copenhagen, 1353 Copenhagen, Denmark

[d] Department of Health Science and Technology, Aalborg University, 9220 Aalborg East, Denmark

[e] UCD Clinical Research Centre, School of Medicine, University College Dublin, Dublin 4, Ireland.

[f] Center for Research in Childhood Health, Department of Regional Health Research, University of Southern Denmark, DK-5230 Odense, Denmark

[g] Head of studies, Education and Social Education Svendborg, UCL University College, 5700 Svendborg, Denmark

[h] Department of Applied Research, University College South (UCSYD), 6100 Haderslev, Denmark

* Corresponding author:
E-Mail address: clykkegaard@health.sdu.dk (C.R. Lykkegaard)
University of Southern Denmark
J.B Winsloews Vej 9
DK-5230 Odense M, Denmark
Mobile phone + 45 30 24 75 86

Supplementary material

In total we had 245,703 observations and 401,955 numbers of sport sessions (Table S1). In girls we had 127867 and in boys 117836 weeks of observations, mean number of sport participations were 1.55 in girls with the lowest and highest being 1.09 (SD 1.06) and 1.75 (SD 1.53) at age 6 to 7 and 12-13 respectively, in boys´ participation lowest and highest means were 1.12 and 1.90 at age 6 to 7 and 12-13 (Table S1).

Table S1 . Descriptive statistics for sports participation over 5 years calculated in numbers of sessions per week divided in age groups.

	Age (years)						
	**6-7**	**8-9**	**10-11**	**12-13**	**14-15**	**16-17**	**All**
**N students with at least one weekly observation in period**	254	797	1351	1291	559	82	1547
**Sports sessions per week**							
Total number of weekly observations	7072	43040	88614	77587	27960	1430	245703
Total number of sports sessions	7496	57865	146963	140356	47563	1712	401955
**Main outcome: Weekly sports participation***							
Total number of monthly observations	1935	14492	36562	34689	11690	416	99784
Median (range) of weekly sport sessions pr. month	1.00 (0-5)	1.20 (0-8)	1.50 (0-8)	1.60 (0-8)	1.50 (0-7)	0.75 (0-6)	1.40 (0-8)
**Girls N**	144	420	704	664	278	40	798
**Sports sessions per week**							
Total number of weekly observations	4049	23662	47116	38760	13676	604	127867
Total number of sports sessions	4258	30568	75682	67252	21591	697	200048
**Main outcome: Weekly sports participation***							
Total number of monthly observations	1092	7624	18844	16616	5294	168	49638

Median (Range) of weekly sport sessions pr. month	1.00 (0-5)	1.00 (0-8)	1.33 (0-8)	1.50 (0-8)	1.25 (0-7)	0.75 (0-6)	1.25 (0-8)
**Boys N**	110	377	647	627	281	42	749
**Sports sessions per week**							
Total number of weekly observations	3023	19378	41498	38827	14284	826	117836
Total number of sports sessions	3238	27297	71281	73104	25972	1015	201907
Median (range) of sport sessions per week*	1.00 (0,8)	1.00 (0,8)	2.00 (0,8)	2.00 (0,8)	2.00 (0,8)	1.00 (0,8)	2.00 (0,8)
**Main outcome: Weekly sports participation***							
Total number of monthly observations	843	6868	17719	18073	6396	248	50146
Median (range) of weekly sport sessions pr. month ****	1.00 (0,4)	1.25 (0,6)	1.60 (0,8)	1.75 (0,8)	1.67 (0,7)	0.75 (0,6)	1.50 (0,8)

* Main outcome: Average weekly sports participation per week per month calculated per child

Table S2. Descriptive statistics for the different sport types over 5 years calculated in numbers of sessions per week divided in age groups.

	Age (years)						
	**6-7**	**8-9**	**10-11**	**12-13**	**14-15**	**16-17**	**All**
**All students** with at least one weekly observation in period	254	797	1351	1291	559	82	1547
*Type of sports sessions per week, n (%)**							
All	7496.0 (100)	57865.0 (100)	146963.0 (100)	140356.0 (100)	47563.0 (100)	1712.0 (100)	401955.0 (100)
Soccer	1772.1 (23.6)	16488.7 (28.5)	43915.6 (29.9)	44509.5 (31.7)	13227.7 (27.8)	312.0 (18.2)	120225.6 (29.9)
Handball	948.5 (12.7)	9380.4 (16.2)	31907.4 (21.7)	32139.8 (22.9)	10844.6 (22.8)	346.0 (20.2)	85566.7 (21.3)
Swimming	1620.0 (21.6)	8591.3 (14.8)	11268.5 (7.7)	6976.1 (5.0)	1409.6 (3.0)	26.7 (1.6)	29892.1 (7.4)
Horseback riding	320.7 (4.3)	3860.3 (6.7)	12685.4 (8.6)	12765.0 (9.09)	4391.7 (9.2)	177.0 (10.3)	34200.1 (8.5)
Rythm gymnastic	708.1 (9.4)	2879.0 (5.0)	4395.3 (3.0)	3371.4 (2.4)	1066.5 (2.2)	51.2 (3.0)	12471.4 (3.1)
Tumbling gymnastic	922.2 (12.3)	5375.5 (9.3)	10372.0 (7.06)	7755.1 (5.5)	1680.0 (3.5)	13.0 (0.8)	26117.8 (6.5)
Basketball	9.5 (0.1)	320.9 (0.6)	1805.8 (1.2)	3280.5 (2.3)	1640.4 (3.4)	142.0 (8.3)	7199.2 (1.8)
Volleyball	39.0 (0.5)	857.4 (1.5)	2487.1 (1.7)	2819.0 (2.01)	1362.7 (2.9)	32.0 (1.9)	7597.2 (1.9)
Dance	346.0 (4.6)	2271.7 (3.9)	3741.9 (2.5)	3161.9 (2.3)	1752.7 (3.7)	71.0 (4.1)	11345.1 (2.8)
Others	757.0 (10.1)	7235.9 (12.5)	22552.3 (15.3)	22089.7 (15.7)	9693.2 (20.4)	532.2 (31.08)	62860.2 (15.6)
**Girls**	144	420	704	664	278	40	798
*Type of sports sessions per week, n (%)**							
All	4258.0 (100)	30568.0 (100)	75682.0 (100)	67252.0 (100)	21591.0 (100)	697.0 (100)	200048.0 (100)
Soccer	529.8 (12.4)	4675.4 (15.3)	11838.4 (15.6)	11886.4 (17.7)	2831.0 (13.1)	0.0 (0.00)	31761.1 (15.9)
Handball	535.4 (12.6)	4982.9 (16.3)	17850.7 (23.6)	15718.8 (23.4)	5728.9 (26.5)	241.0 (34.6)	45057.7 (22.5)
Swimming	903.8 (21.2)	4775.7 (15.6)	6209.4 (8.2)	4222.4 (6.3)	564.9 (2.6)	5.7 (0.8)	16681.9 (8.3)
Horseback riding	320.7 (7.5)	3818.3 (12.5)	12250.1 (16.2)	12277.7 (18.3)	4371.7 (20.2)	177.0 (25.4)	33215.4 (16.6)
Rythm gymnastic	530.8 (12.5)	2280.2 (7.5)	3748.4 (5.0)	3000.3 (4.5)	994.5 (4.6)	50.2 (7.2)	10604.2 (5.3)
Tumbling gymnastic	740.3 (17.4)	3842.4 (12.6)	7250.1 (9.6)	5464.4 (8.1)	881.0 (4.08)	8.0 (1.1)	18186.2 (9.09)
Basketball	5.5 (0.1)	76.1 (0.2)	370.2 (0.5)	411.4 (0.6)	178.2 (0.8)	3.0 (0.4)	1044.4 (0.5)
Volleyball	7.5 (0.2)	485.5 (1.6)	1628.2 (2.2)	1686.3 (2.5)	883.2 (4.09)	30.0 (4.3)	4720.8 (2.4)
Dance	345.0 (8.1)	2195.4 (7.2)	3583.2 (4.7)	3038.0 (4.5)	1497.8 (6.9)	71.0 (10.2)	10730.4 (5.4)
Others	308.3 (7.2)	3060.2 (10.01)	9779.4 (12.9)	8716.3 (13.0)	3432.7 (15.9)	108.2 (15.5)	25405.0 (12.7)
**Boys**	110	377	647	627	281	42	749
*Type of sports sessions per week, n (%)**							
All	3238.0 (100)	27297.0 (100)	71281.0 (100)	73104.0 (100)	25972.0 (100)	1015.0 (100)	201907.0 (100)
Soccer	1242.3 (38.4)	11813.3 (43.3)	32077.3 (45.00)	32623.1 (44.6)	10396.7 (40.03)	312.0 (30.7)	88464.5 (43.8)
Handball	413.1 (12.8)	4397.5 (16.1)	14056.8 (19.7)	16421.0 (22.5)	5115.7 (19.7)	105.0 (10.3)	40509.0 (20.06)

Swimming	716.3 (22.1)	3815.6 (14.0)	5059.1 (7.1)	2753.7 (3.8)	844.7 (3.3)	21.0 (2.07)	13210.3 (6.5)
Horseback riding	0.0 (0.00)	42.0 (0.2)	435.3 (0.6)	487.3 (0.7)	20.0 (0.08)	0.0 (0.00)	984.7 (0.5)
Rythm gymnastic	177.3 (5.5)	598.8 (2.2)	646.9 (0.9)	371.2 (0.5)	72.0 (0.3)	1.0 (0.1)	1867.2 (0.9)
Tumbling gymnastic	181.8 (5.6)	1533.2 (5.6)	3121.9 (4.4)	2290.7 (3.1)	799.0 (3.08)	5.0 (0.5)	7931.6 (3.9)
Basketball	4.0 (0.1)	244.8 (0.9)	1435.7 (2.01)	2869.1 (3.9)	1462.2 (5.6)	139.0 (13.7)	6154.7 (3.05)
Volleyball	31.5 (1.0)	371.9 (1.4)	858.8 (1.2)	1132.7 (1.5)	479.5 (1.8)	2.0 (0.2)	2876.4 (1.4)
Dance	1.0 (0.03)	76.3 (0.3)	158.8 (0.2)	123.9 (0.2)	254.8 (1.0)	0.0 (0.00)	614.8 (0.3)
Others	448.8 (13.9)	4175.7 (15.3)	12772.8 (17.9)	13373.4 (18.3)	6260.5 (24.1)	424.0 (41.8)	37455.2 (18.6)

* If more than one sports type was reported and the reported number of sessions exceeded the number of reported sport types, the reported number of sport sessions were equally allocated between the reported sport types.

Table S3. Descriptive statistics for the students' motor performance baseline z-scores divided in low, middle, and high by age groups.

Motor performance score at baseline	Age (years)			
	**6-7**	**8-9**	**10-11**	**12-13**
**All students** N				
**Total motor performance[A] z-score***				
Low	-1.5 - -0.3	-2.3 - -0.3	-2.5 - -0.2	-1.1 - -0.4
Middle	-0.3 - 0.2	-0.3 - 0.3	-0.2 - 0.3	-0.4 - 0.2
High	0.3 - 1.8	0.3 - 1.7	0.3 - 1.8	0.4 - 1.3
**Health-related motor performance[B] z-score***				
Low	-1.7 - -0.4	-2.0 - -0.3	-2.8 - -0.2	-1.5 - -0.2
Middle	-0.4 - 0.4	-0.3 - 0.3	-0.2 - 0.3	-0.1 - 0.3
High	0.4 - 1.9	0.3 - 3.0	0.3 - 2.3	0.4 - 1.1
**Coordination-related motor performance[C] z-score***				
Low	-1.6 - -0.3	-2.6 - -0.3	-2.4 - -0.2	-0.9 - -0.3
Middle	-0.3 - 0.3	-0.3 - 0.3	-0.2 - 0.3	-0.3 - 0.3
High	0.3 - 1.8	0.3 - 1.8	0.3 - 1.9	0.3 - 1.5
**Girls N**				
**Total motor performance[A] z-score***				
Low	-1.2 - -0.3	-1.7 - -0.3	-1.6 - -0.2	-1.1 - -0.4
Middle	-0.3 - 0.2	-0.3 - 0.3	-0.2 - 0.3	-0.0 - 0.4
High	0.2 - 1.8	0.3 - 1.7	0.3 - 1.8	0.4 - 0.7
**Health-related motor performance[B] z-score***				
Low	-1.4 - -0.4	-1.9 - -0.3	-1.8 - -0.3	-1.5 - -0.3
Middle	-0.4 - 0.4	-0.3 - 0.3	-0.3 - 0.3	-0.1 - 0.4
High	0.4 - 1.9	0.3 - 3.0	0.3 - 2.1	0.4 - 1.1
**Coordination-related motor performance[C] z-score***				
Low	-1.2 - -0.3	-2.3 - -0.3	-2.0 - -0.2	-0.8 - -0.3
Middle	-0.3 - 0.2	-0.3 - 0.3	-0.2 - 0.3	-0.3 - 0.3
High	0.2 - 1.7	0.3 - 1.8	0.3 - 1.9	0.3 - 1.0
**Boys N**				
**Total motor performance[A] z-score***				
Low	-1.5 - -0.2	-2.3 - -0.3	-2.5 - -0.2	-0.6 - -0.4
Middle	-0.2 - 0.3	-0.3 - 0.3	-0.2 - 0.2	-0.4 - 0.1
High	0.3 - 1.5	0.3 - 1.7	0.2 - 1.7	0.2 - 1.3
**Health-related motor performance[B] z-score***				

Low	-1.7 - -0.4	-2.0 - -0.3	-2.8 - -0.2	-0.7 - -0.2
Middle	-0.3 - 0.3	-0.3 - 0.3	-0.2 - 0.2	-0.1 - 0.2
High	0.3 - 1.6	0.3 - 1.8	0.2 - 2.3	0.3 - 1.0
**Coordination-related motor performance[C] z-score***				
Low	-1.6 - -0.3	-2.6 - -0.4	-2.4 - -0.2	-0.9 - -0.5
Middle	-0.3 - 0.3	-0.3 - 0.3	-0.2 - 0.3	-0.2 - 0.3
High	0.3 - 1.8	0.3 - 1.7	0.3 - 1.5	0.6 - 1.5

* Z-score = (variable value – mean of values)/SD, where mean and SD is with respect to the gender and age of the participant which z-scores is being calculated

[A] Including all 6 motor performance tests

[B] Including the hand grip test and the Andersen test

[C] Including vertical jump, shuttle run, backward balance, and precision throw

Table S4. Results for group-based trajectory models for sports participation over (monthly) age, based on 1547 children with a total of 61,398 monthly observations. Model 4G was fitted for four groups, model 5G for five and model 6G for six groups.

Sports participation trajectory groups*	Cumulative probability of belonging to group**	Assigned membership based on maximum rule N (%)	APPA[a]	OCC[b]
**Model 4G**				
Group 1	24.9	382 (24.7)	0.98	135.9
Group 2	37.2	580 (37.5)	0.96	40.3
Group 3	28.0	433 (28.0)	0.97	86.6
Group 4	9.9	152 (9.8)	0.99	1293.7
**Model 5G**				
Group 1	13.7	209 (13.5)	0.98	337.9
Group 2	26.5	417 (27.0)	0.94	46.1
Group 3	29.2	448 (29.0)	0.95	43.9
Group 4	22.0	340 (22.0)	0.96	86.1
Group 5	8.6	133 (8.6)	0.98	529.6
**Model 6G**				
Group 1	13.6	210 (13.6)	0.98	316.5
Group 2	26.1	409 (26.4)	0.94	46.8
Group 3	28.7	441 (28.5)	0.94	42.7
Group 4	21.5	334 (21.6)	0.96	88.4
Group 5	9.2	139 (9.0)	0.99	1005.6
Group 6	0.9	14 (0.9)	0.99	7961.0

* Group 1 is the subgroup of individuals with the lowest activity level in all three models, and group 4/5/6 with the highest activity level in model 4/5/6 respectively

** regardless of group assignment per maximum rule

[a] APPA = Average Posterior Probability of Assignment

[b] OCC = Odds of correct classification

Table S5. Estimated expected group-trajectories by (monthly) age and 95% confidence interval (CI, in parentheses) for model 5G.

		Model 5G				
**Age (in years)**	**Monthly age**	**Group 1**	**Group 2**	**Group 3**	**Group 4**	**Group 5**
6	72		0.815 (0.603-1.028)			
	73		0.815 (0.614-1.016)			
	74		0.815 (0.625-1.005)			
	75	0.267 (0.241-0.293)	0.815 (0.635-0.995)			
	76	0.275 (0.245-0.304)	0.815 (0.645-0.986)			1.145 (0.777-1.513)
	77	0.282 (0.250-0.313)	0.816 (0.655-0.977)		1.658 (1.421-1.894)	1.205 (0.853-1.556)
	78	0.289 (0.255-0.322)	0.816 (0.664-0.968)	1.007 (0.802-1.212)	1.651 (1.426-1.875)	1.265 (0.931-1.599)
	79	0.295 (0.260-0.330)	0.817 (0.673-0.960)	1.021 (0.826-1.216)	1.645 (1.433-1.858)	1.326 (1.010-1.642)
	80	0.301 (0.266-0.337)	0.818 (0.682-0.953)	1.035 (0.849-1.220)	1.641 (1.440-1.842)	1.387 (1.089-1.685)
	81	0.307 (0.271-0.344)	0.819 (0.691-0.946)	1.049 (0.872-1.225)	1.638 (1.448-1.828)	1.448 (1.168-1.728)
	82	0.313 (0.276-0.349)	0.820 (0.699-0.940)	1.063 (0.895-1.230)	1.636 (1.456-1.816)	1.510 (1.248-1.771)
	83	0.318 (0.282-0.354)	0.821 (0.708-0.934)	1.076 (0.918-1.235)	1.636 (1.466-1.806)	1.571 (1.327-1.815)
7	84	0.323 (0.287-0.359)	0.822 (0.715-0.929)	1.090 (0.940-1.241)	1.636 (1.476-1.797)	1.633 (1.405-1.860)
	85	0.327 (0.292-0.362)	0.823 (0.723-0.924)	1.104 (0.962-1.247)	1.638 (1.487-1.789)	1.694 (1.483-1.905)
	86	0.331 (0.297-0.365)	0.825 (0.730-0.919)	1.118 (0.983-1.253)	1.641 (1.498-1.784)	1.756 (1.560-1.951)
	87	0.335 (0.302-0.368)	0.826 (0.737-0.916)	1.132 (1.004-1.260)	1.645 (1.510-1.779)	1.817 (1.635-1.998)
	88	0.338 (0.306-0.370)	0.828 (0.744-0.912)	1.146 (1.025-1.267)	1.650 (1.523-1.777)	1.878 (1.710-2.046)
	89	0.341 (0.310-0.372)	0.830 (0.751-0.909)	1.160 (1.046-1.275)	1.656 (1.536-1.775)	1.938 (1.783-2.094)
	90	0.344 (0.314-0.373)	0.832 (0.757-0.907)	1.174 (1.066-1.282)	1.663 (1.550-1.775)	1.999 (1.855-2.142)
	91	0.346 (0.317-0.374)	0.833 (0.763-0.904)	1.188 (1.085-1.290)	1.670 (1.564-1.777)	2.059 (1.926-2.191)
	92	0.347 (0.320-0.375)	0.835 (0.768-0.903)	1.202 (1.104-1.299)	1.679 (1.579-1.780)	2.118 (1.995-2.241)
	93	0.349 (0.323-0.375)	0.837 (0.774-0.901)	1.215 (1.123-1.308)	1.689 (1.594-1.784)	2.177 (2.063-2.291)
	94	0.350 (0.325-0.375)	0.839 (0.779-0.900)	1.229 (1.142-1.317)	1.700 (1.609-1.790)	2.236 (2.130-2.342)
	95	0.350 (0.326-0.375)	0.841 (0.783-0.899)	1.243 (1.159-1.326)	1.711 (1.626-1.797)	2.294 (2.195-2.392)
8	96	0.351 (0.328-0.374)	0.843 (0.788-0.899)	1.256 (1.177-1.335)	1.723 (1.642-1.805)	2.351 (2.259-2.443)
	97	0.351 (0.328-0.373)	0.846 (0.792-0.899)	1.270 (1.194-1.345)	1.736 (1.659-1.814)	2.408 (2.321-2.494)
	98	0.350 (0.329-0.372)	0.848 (0.796-0.899)	1.283 (1.211-1.355)	1.750 (1.677-1.824)	2.464 (2.383-2.546)
	99	0.350 (0.329-0.370)	0.850 (0.800-0.900)	1.296 (1.227-1.366)	1.765 (1.694-1.836)	2.520 (2.443-2.597)

	100	0.349 (0.329-0.369)	0.852 (0.803-0.901)	1.309 (1.243-1.376)	1.780 (1.713-1.848)	2.575 (2.502-2.648)
	101	0.347 (0.328-0.367)	0.854 (0.807-0.901)	1.323 (1.258-1.387)	1.797 (1.731-1.862)	2.629 (2.559-2.699)
	102	0.346 (0.327-0.365)	0.856 (0.810-0.902)	1.336 (1.273-1.398)	1.813 (1.750-1.876)	2.683 (2.616-2.750)
	103	0.344 (0.325-0.362)	0.858 (0.813-0.904)	1.348 (1.288-1.409)	1.831 (1.770-1.892)	2.736 (2.671-2.800)
	104	0.342 (0.324-0.359)	0.860 (0.816-0.905)	1.361 (1.302-1.420)	1.849 (1.789-1.908)	2.788 (2.726-2.851)
	105	0.339 (0.322-0.356)	0.862 (0.818-0.906)	1.374 (1.316-1.431)	1.867 (1.810-1.925)	2.840 (2.779-2.901)
	106	0.336 (0.320-0.353)	0.864 (0.821-0.907)	1.386 (1.330-1.443)	1.887 (1.830-1.943)	2.891 (2.831-2.950)
	107	0.334 (0.317-0.350)	0.866 (0.823-0.909)	1.398 (1.343-1.454)	1.906 (1.851-1.961)	2.941 (2.883-2.999)
9	108	0.330 (0.315-0.346)	0.868 (0.826-0.910)	1.410 (1.356-1.465)	1.927 (1.873-1.980)	2.990 (2.933-3.047)
	109	0.327 (0.312-0.342)	0.870 (0.828-0.911)	1.422 (1.369-1.476)	1.947 (1.895-2.000)	3.039 (2.983-3.095)
	110	0.323 (0.309-0.338)	0.872 (0.831-0.912)	1.434 (1.381-1.487)	1.969 (1.917-2.020)	3.087 (3.031-3.142)
	111	0.320 (0.306-0.333)	0.873 (0.833-0.913)	1.446 (1.394-1.498)	1.990 (1.940-2.041)	3.134 (3.079-3.189)
	112	0.316 (0.303-0.329)	0.875 (0.835-0.914)	1.457 (1.406-1.509)	2.012 (1.963-2.062)	3.180 (3.126-3.234)
	113	0.312 (0.299-0.324)	0.876 (0.837-0.915)	1.468 (1.418-1.519)	2.035 (1.986-2.083)	3.226 (3.173-3.279)
	114	0.308 (0.296-0.319)	0.878 (0.840-0.916)	1.479 (1.429-1.530)	2.057 (2.009-2.105)	3.271 (3.218-3.323)
	115	0.303 (0.292-0.314)	0.879 (0.842-0.916)	1.490 (1.441-1.540)	2.080 (2.033-2.127)	3.315 (3.263-3.367)
	116	0.299 (0.289-0.309)	0.880 (0.844-0.917)	1.501 (1.452-1.550)	2.104 (2.057-2.150)	3.358 (3.306-3.409)
	117	0.294 (0.285-0.304)	0.881 (0.846-0.917)	1.511 (1.463-1.560)	2.127 (2.082-2.172)	3.400 (3.349-3.451)
	118	0.289 (0.281-0.298)	0.882 (0.848-0.917)	1.521 (1.473-1.570)	2.151 (2.107-2.195)	3.442 (3.391-3.492)
	119	0.285 (0.277-0.293)	0.883 (0.849-0.916)	1.531 (1.484-1.579)	2.175 (2.131-2.218)	3.482 (3.433-3.532)
10	120	0.280 (0.273-0.287)	0.884 (0.851-0.916)	1.541 (1.494-1.588)	2.199 (2.156-2.242)	3.522 (3.473-3.571)
	121	0.275 (0.269-0.282)	0.884 (0.853-0.915)	1.551 (1.504-1.597)	2.223 (2.181-2.265)	3.561 (3.513-3.610)
	122	0.270 (0.264-0.276)	0.884 (0.855-0.914)	1.560 (1.513-1.606)	2.247 (2.207-2.288)	3.600 (3.552-3.647)
	123	0.265 (0.260-0.270)	0.885 (0.856-0.913)	1.569 (1.522-1.615)	2.272 (2.232-2.312)	3.637 (3.590-3.684)
	124	0.260 (0.256-0.264)	0.885 (0.858-0.912)	1.577 (1.532-1.623)	2.296 (2.257-2.335)	3.673 (3.627-3.720)
	125	0.255 (0.251-0.259)	0.884 (0.859-0.910)	1.586 (1.540-1.631)	2.321 (2.282-2.359)	3.709 (3.663-3.755)
	126	0.250 (0.247-0.253)	0.884 (0.860-0.908)	1.594 (1.549-1.639)	2.345 (2.307-2.382)	3.744 (3.698-3.789)
	127	0.245 (0.243-0.247)	0.883 (0.862-0.905)	1.602 (1.557-1.647)	2.369 (2.332-2.406)	3.778 (3.733-3.823)
	128	0.240 (0.238-0.242)	0.883 (0.863-0.903)	1.609 (1.564-1.654)	2.393 (2.357-2.429)	3.811 (3.766-3.855)
	129	0.235 (0.234-0.236)	0.882 (0.864-0.900)	1.616 (1.572-1.661)	2.417 (2.382-2.453)	3.843 (3.799-3.887)
	130	0.230 (0.230-0.231)	0.881 (0.865-0.896)	1.623 (1.579-1.668)	2.441 (2.406-2.476)	3.874 (3.830-3.918)
	131	0.225 (0.225-0.225)	0.879 (0.866-0.892)	1.630 (1.585-1.675)	2.465 (2.431-2.499)	3.905 (3.861-3.948)
11	132	0.220 (0.220-0.221)	0.878 (0.868-0.887)	1.636 (1.591-1.681)	2.488 (2.455-2.522)	3.934 (3.890-3.977)
	133	0.215 (0.214-0.217)	0.876 (0.871-0.880)	1.642 (1.597-1.687)	2.512 (2.478-2.545)	3.962 (3.919-4.006)
	134	0.211 (0.209-0.213)	0.874 (0.874-0.874)	1.648 (1.603-1.693)	2.535 (2.502-2.568)	3.990 (3.947-4.034)
	135	0.206 (0.203-0.209)	0.871 (0.871-0.871)	1.653 (1.608-1.699)	2.557 (2.524-2.590)	4.017 (3.973-4.060)

	136	0.201 (0.198-0.205)	0.869 (0.869-0.869)	1.658 (1.612-1.704)	2.579 (2.547-2.612)	4.042 (3.999-4.086)
	137	0.197 (0.193-0.201)	0.866 (0.866-0.866)	1.663 (1.616-1.709)	2.601 (2.569-2.634)	4.067 (4.023-4.112)
	138	0.192 (0.188-0.197)	0.863 (0.863-0.863)	1.667 (1.620-1.714)	2.623 (2.590-2.655)	4.091 (4.046-4.136)
	139	0.188 (0.183-0.193)	0.859 (0.859-0.859)	1.671 (1.623-1.718)	2.644 (2.611-2.676)	4.114 (4.069-4.159)
	140	0.184 (0.178-0.189)	0.856 (0.856-0.856)	1.674 (1.626-1.722)	2.664 (2.632-2.697)	4.136 (4.090-4.182)
	141	0.179 (0.173-0.186)	0.852 (0.852-0.852)	1.678 (1.629-1.726)	2.684 (2.651-2.717)	4.157 (4.111-4.204)
	142	0.175 (0.168-0.182)	0.848 (0.848-0.848)	1.680 (1.631-1.730)	2.704 (2.670-2.737)	4.177 (4.130-4.224)
	143	0.171 (0.164-0.179)	0.843 (0.843-0.843)	1.683 (1.632-1.733)	2.723 (2.689-2.756)	4.196 (4.148-4.244)
12	144	0.167 (0.159-0.176)	0.838 (0.838-0.838)	1.685 (1.634-1.736)	2.741 (2.707-2.775)	4.214 (4.165-4.263)
	145	0.164 (0.155-0.173)	0.833 (0.833-0.833)	1.686 (1.634-1.738)	2.758 (2.724-2.793)	4.231 (4.182-4.281)
	146	0.160 (0.150-0.169)	0.828 (0.828-0.828)	1.688 (1.635-1.741)	2.775 (2.741-2.810)	4.247 (4.197-4.298)
	147	0.156 (0.146-0.167)	0.822 (0.822-0.822)	1.688 (1.635-1.742)	2.792 (2.756-2.827)	4.263 (4.211-4.314)
	148	0.153 (0.142-0.164)	0.816 (0.816-0.816)	1.689 (1.634-1.744)	2.807 (2.771-2.843)	4.277 (4.224-4.329)
	149	0.150 (0.138-0.161)	0.810 (0.810-0.810)	1.689 (1.633-1.745)	2.822 (2.786-2.858)	4.290 (4.237-4.343)
	150	0.146 (0.134-0.158)	0.803 (0.803-0.803)	1.688 (1.632-1.745)	2.836 (2.799-2.873)	4.302 (4.248-4.356)
	151	0.143 (0.130-0.156)	0.796 (0.796-0.796)	1.688 (1.630-1.745)	2.849 (2.812-2.887)	4.313 (4.258-4.368)
	152	0.140 (0.127-0.154)	0.789 (0.789-0.789)	1.686 (1.627-1.745)	2.862 (2.823-2.900)	4.323 (4.267-4.379)
	153	0.137 (0.123-0.151)	0.781 (0.781-0.781)	1.685 (1.625-1.744)	2.873 (2.834-2.912)	4.332 (4.275-4.389)
	154	0.135 (0.120-0.149)	0.774 (0.774-0.774)	1.682 (1.622-1.743)	2.884 (2.844-2.923)	4.340 (4.283-4.398)
	155	0.132 (0.117-0.147)	0.765 (0.765-0.765)	1.680 (1.618-1.741)	2.893 (2.853-2.933)	4.347 (4.289-4.405)
13	156	0.129 (0.114-0.145)	0.757 (0.757-0.757)	1.677 (1.614-1.739)	2.902 (2.861-2.942)	4.353 (4.294-4.412)
	157	0.127 (0.111-0.143)	0.748 (0.748-0.748)	1.673 (1.609-1.737)	2.909 (2.868-2.950)	4.358 (4.298-4.417)
	158	0.125 (0.108-0.142)	0.739 (0.739-0.739)	1.669 (1.604-1.734)	2.916 (2.874-2.957)	4.362 (4.301-4.422)
	159	0.122 (0.105-0.140)	0.729 (0.729-0.729)	1.665 (1.599-1.730)	2.921 (2.879-2.964)	4.364 (4.303-4.425)
	160	0.120 (0.102-0.138)	0.719 (0.719-0.719)	1.660 (1.593-1.726)	2.926 (2.883-2.968)	4.366 (4.304-4.428)
	161	0.118 (0.100-0.137)	0.709 (0.709-0.709)	1.654 (1.587-1.722)	2.929 (2.885-2.972)	4.367 (4.304-4.429)
	162	0.117 (0.098-0.136)	0.699 (0.699-0.699)	1.648 (1.580-1.717)	2.931 (2.887-2.975)	4.366 (4.303-4.429)
	163	0.115 (0.096-0.134)	0.688 (0.688-0.688)	1.642 (1.572-1.712)	2.932 (2.887-2.977)	4.364 (4.301-4.428)
	164	0.113 (0.093-0.133)	0.677 (0.677-0.677)	1.635 (1.564-1.706)	2.931 (2.886-2.977)	4.362 (4.297-4.426)
	165	0.112 (0.091-0.132)	0.665 (0.665-0.665)	1.628 (1.556-1.700)	2.930 (2.884-2.976)	4.358 (4.293-4.423)
	166	0.111 (0.090-0.131)	0.654 (0.654-0.654)	1.620 (1.547-1.693)	2.927 (2.880-2.974)	4.353 (4.287-4.419)
	167	0.109 (0.088-0.131)	0.642 (0.642-0.642)	1.612 (1.537-1.686)	2.923 (2.875-2.971)	4.347 (4.279-4.415)
14	168	0.108 (0.086-0.130)	0.629 (0.629-0.629)	1.603 (1.527-1.679)	2.917 (2.868-2.966)	4.340 (4.271-4.409)
	169	0.107 (0.085-0.130)	0.617 (0.617-0.617)	1.593 (1.516-1.671)	2.910 (2.860-2.960)	4.332 (4.261-4.402)
	170	0.106 (0.083-0.130)	0.604 (0.604-0.604)	1.584 (1.504-1.663)	2.902 (2.850-2.953)	4.322 (4.249-4.395)
	171	0.106 (0.082-0.130)	0.591 (0.591-0.591)	1.573 (1.492-1.655)	2.892 (2.839-2.945)	4.312 (4.237-4.387)

	172	0.105 (0.080-0.130)	0.577 (0.577-0.577)	1.562 (1.479-1.646)	2.881 (2.826-2.935)	4.300 (4.222-4.378)
	173	0.105 (0.079-0.130)	0.564 (0.564-0.564)	1.551 (1.465-1.637)	2.868 (2.812-2.924)	4.287 (4.206-4.368)
	174	0.104 (0.078-0.131)	0.550 (0.550-0.550)	1.539 (1.450-1.628)	2.854 (2.795-2.912)	4.273 (4.189-4.357)
	175	0.104 (0.076-0.132)	0.536 (0.536-0.536)	1.527 (1.435-1.618)	2.838 (2.777-2.899)	4.258 (4.170-4.346)
	176	0.104 (0.075-0.133)	0.522 (0.522-0.522)	1.514 (1.419-1.609)	2.820 (2.757-2.884)	4.242 (4.149-4.334)
	177	0.104 (0.074-0.134)	0.508 (0.500-0.515)	1.500 (1.402-1.599)	2.801 (2.735-2.868)	4.224 (4.127-4.322)
	178	0.104 (0.073-0.136)	0.493 (0.479-0.507)	1.486 (1.384-1.589)	2.781 (2.711-2.850)	4.206 (4.103-4.309)
	179	0.105 (0.072-0.138)	0.478 (0.460-0.497)	1.472 (1.365-1.578)	2.758 (2.685-2.832)	4.186 (4.077-4.295)
15	180	0.105 (0.070-0.140)	0.464 (0.442-0.486)	1.457 (1.345-1.568)	2.734 (2.656-2.812)	4.165 (4.049-4.280)
	181	0.106 (0.069-0.142)	0.449 (0.424-0.473)	1.441 (1.325-1.558)	2.708 (2.626-2.790)	4.142 (4.020-4.265)
	182	0.106 (0.068-0.145)	0.434 (0.407-0.461)	1.425 (1.303-1.547)	2.681 (2.594-2.767)	4.119 (3.989-4.249)
	183	0.107 (0.068-0.147)	0.419 (0.390-0.447)	1.408 (1.281-1.536)	2.651 (2.559-2.743)	4.094 (3.956-4.233)
	184	0.109 (0.067-0.150)	0.403 (0.374-0.433)	1.391 (1.257-1.525)	2.620 (2.522-2.718)	4.068 (3.921-4.215)
	185	0.110 (0.066-0.154)	0.388 (0.358-0.419)	1.374 (1.233-1.514)	2.587 (2.483-2.691)	4.041 (3.885-4.198)
	186	0.112 (0.066-0.157)	0.373 (0.342-0.404)	1.355 (1.208-1.503)	2.552 (2.441-2.663)	4.013 (3.847-4.179)
	187	0.113 (0.065-0.161)	0.358 (0.328-0.389)	1.337 (1.181-1.492)	2.515 (2.397-2.633)	3.983 (3.807-4.160)
	188	0.115 (0.065-0.165)	0.343 (0.314-0.373)	1.318 (1.154-1.481)	2.476 (2.351-2.602)	3.953 (3.765-4.141)
	189	0.117 (0.065-0.169)	0.328 (0.300-0.356)	1.298 (1.126-1.470)	2.436 (2.302-2.570)	3.921 (3.721-4.120)
	190	0.120 (0.066-0.174)	0.313 (0.287-0.339)	1.278 (1.097-1.459)	2.393 (2.250-2.536)	3.887 (3.676-4.099)
	191	0.122 (0.067-0.178)	0.298 (0.275-0.322)	1.257 (1.067-1.447)	2.348 (2.196-2.501)	3.853 (3.628-4.078)
16	192	0.125 (0.068-0.183)	0.284 (0.264-0.304)	1.236 (1.037-1.436)	2.302 (2.139-2.465)	3.817 (3.579-4.055)
	193	0.129 (0.069-0.188)	0.269 (0.253-0.286)	1.215 (1.005-1.424)	2.253 (2.079-2.427)	3.780 (3.527-4.032)
	194	0.132 (0.072-0.193)	0.255 (0.244-0.267)	1.192 (0.973-1.412)	2.203 (2.017-2.388)	3.742 (3.474-4.009)
	195	0.136 (0.075-0.198)	0.241 (0.235-0.248)	1.170 (0.940-1.400)	2.150 (1.951-2.348)	3.702 (3.419-3.985)
	196	0.140 (0.078-0.202)	0.228 (0.227-0.228)	1.147 (0.906-1.388)	2.095 (1.883-2.307)	3.661 (3.362-3.960)
	197	0.145 (0.082-0.207)	0.214 (0.208-0.220)	1.124 (0.872-1.375)	2.039 (1.812-2.265)	
	198	0.150 (0.088-0.212)	0.201 (0.188-0.214)	1.100 (0.837-1.363)		
	199		0.188 (0.167-0.209)			
	200		0.176 (0.147-0.205)			
	201		0.164 (0.126-0.201)			
